# Feasibility and efficiency of concurrent chemoradiotherapy with a single agent or double agents vs radiotherapy alone for elderly patients with esophageal squamous cell carcinoma: Experience of two centers

**DOI:** 10.1002/cam4.1788

**Published:** 2019-01-01

**Authors:** Chunyue Huang, Yujia Zhu, Qiwen Li, Wenwen Zhang, Hui Liu, Weijun Zhang, Yonghong Hu, Yawei Yuan, Mengzhong Liu

**Affiliations:** ^1^ Department of Radiation Oncology Affiliated Cancer Hospital & Institute of Guangzhou Medical University Guangzhou China; ^2^ Department of Radiation Oncology, State Key Laboratory of Oncology in Southern China, Collaborative Innovation Center for Cancer Medicine Sun Yat‐sen University Cancer Center Guangzhou China

**Keywords:** concurrent chemoradiotherapy, elderly patient, overall survival, progression‐free survival, toxicities

## Abstract

The incidence of elderly patients with esophageal cancer (OC) is increasing as the population ages. Until now, the treatment strategy in these patients has been unclear. The aim of our study was to assess the efficiency and tolerance of treatment with radiotherapy alone (RT alone), single‐agent‐based concurrent chemoradiotherapy (CCRT‐1), or double‐agent‐based concurrent chemoradiotherapy (CCRT‐2) in elderly patients (≥65 years) with OC. A total of 271 patients with OC aged 65 years or older were included in this study. The median overall survival (OS), median progression‐free survival (PFS), overall response rate (ORR), disease control rate (DCR), and treatment‐related toxicities were assessed. The median OS time for all patients was 23.6 ± 2.3 months, with 2‐year survival rates of 48.0 ± 3.0%. The median PFS time was 13.6 ± 1.3 months with the 2‐year PFS rate was 33.0 ± 4.0%. Among patients who received CCRT‐1, better OS, and PFS were found in patients who received docetaxel than in patients received fluorouracil and platinum. In a subgroup analysis, 118 patients who underwent RT alone had a median OS time of 15.6 ± 1.9 months and median PFS time of 10.4 ± 0.9 months. The median OS time of patients who received CCRT‐1 was 28.8 ± 10.1 months compared with 27.8 ± 2.5 months for the patients treated with CCRT‐2 (*P *= 0.537). The similar results were observed for median PFS, with 16.5 ± 3.2 months in the CCRT‐1 group and 17.0 ± 2.0 months in the CCRT‐2 group (*P* = 0.321). Grade ≥3 leukocytopenia and grade ≥2 weight loss during treatment occurred in 40.6% and 17.9% of patients, respectively, in the CCRT‐2 group, which was higher than that observed in the CCRT‐1 group. Our results suggested that CCRT could be considered as an acceptable treatment for elderly patients with OC. The CCRT‐1 group presented with a lower incidence of treatment toxicities but comparable survival outcomes, compared to the CCRT‐2 group. Docetaxel was superior to fluorouracil and platinum in terms of OS.

## 1 INTRODUCTION

The incidence of cancer is associated with aging.^1^ In China, esophageal cancer (OC) is the third and fourth most frequent cancer in males and females, respectively, and approximately 69.8% of OC in males occurs in patients older than 60 years.^2^ Moreover, OC patients over the age of 60 years accounted for 44% of all OC cases in the USA^3^ and 54% of all cases in France.^4^ Unfortunately, the therapeutic strategy for elderly patients with OC remains a subject of debate.

Traditionally, elderly patients with OC have been treated with supportive care rather than intensive treatment (radiotherapy, chemoradiotherapy, or surgery) because they were less frequently referred to cancer specialists, and they had multiple comorbidities and poor survival.^5^ However, a retrospective analysis^6^ of elderly patients with OC revealed that patients could benefit from intensive treatment, with a median OS time of 17.8 months in the curative treatment group compared with 5.5 months in the supportive care group. Meanwhile, survival benefits and severe adverse effects (AEs) in elderly patients treated with chemotherapy were similar to those in younger patients,^7^, ^8^ except for a slight increase in the incidence of high‐grade pulmonary toxicity.^8^ At 75 years of age, life expectancy is more than 10 years.^9^ Therefore, elderly patients should not be excluded from intensive treatment based on age alone, and treatment guidelines for these patients are needed.

In our retrospective study, we reviewed two institutional experiences of 271 elderly patients with esophageal squamous cell cancer treated with radiotherapy with or without chemotherapy. Our data suggested that concurrent chemoradiotherapy (CCRT) could be considered as an acceptable treatment for elderly patients with OC. Moreover, compared with the double‐agent‐based concurrent chemoradiotherapy (CCRT‐2) group, the single‐agent‐based concurrent chemoradiotherapy (CCRT‐1) group presented with a lower incidence of treatment toxicities but comparable survival outcomes. In patients treated with CCRT‐1, docetaxel was superior to fluorouracil and platinum in terms of OS but not in terms of PFS. We defined the elderly population based on Social Security and Medicare regulations as persons aged 65 years or older.

## 2 PATIENTS AND METHODS

### 2.1 Patients

We reviewed the records of elderly (≥65 years) patients who were diagnosed with squamous OC and received radiotherapy with or without chemotherapy at Sun Yat‐sen University Cancer Center and Affiliated Cancer Hospital ＆ Institute of Guangzhou Medical University between January 2001 and December 2016. Patients were excluded if they presented with the following: (a) multiple primary esophageal carcinoma; (b) chemotherapy or surgery before/after radiotherapy; (c) a total radiation of dose <40 Gy; (d) postoperative recurrence; (e) OC accompanied by other tumors; or (f) a Karnofsky performance status (KPS) <70.

### 2.2 Clinical characteristics

Gender, age, KPS, body mass index (BMI), smoking status, comorbidities, family history of cancer, tumor length, tumor location, and tumor TNM stage, as well as the radiotherapy techniques applied, tumor early response, and AEs were collected. The tumor TNM stage was determined based on barium esophagography, chest and abdominal computed tomography (CT) scan, and esophageal ultrasonography when it was feasible. Tumors were staged according to the sixth edition of the American Joint Committee on Cancer (AJCC) staging manual.

### 2.3 Radiotherapy

2‐dimensional radiotherapy (2D‐RT), 3‐dimensional conformal radiotherapy (3D‐CRT), or intensity modulated radiotherapy (IMRT) was used in the patients. The mean dose of radiation was 58.4 ± 6.4 Gy (range 40‐74 Gy), and 92.6% of patients completed the radiotherapy.

### 2.4 Chemotherapy

Concurrent chemoradiotherapy was performed in 153 patients. Of these patients, 57 received CCRT‐1, and 96 received CCRT‐2. Commonly used single agents included fluorouracil (n = 19), platinum (n = 27), and docetaxel (n = 11). A total of 96 patients received platinum‐based chemotherapy combined with fluorouracil (n = 55) or paclitaxel/docetaxel (n = 41).

### 2.5 Response, toxicity, survival, and recurrence

Early responses were evaluated by barium esophagography or chest and abdominal CT scan at the end of treatment, and the responses were classified according to Eisenhauer's report.^10^ Acute toxicities were graded according to the Radiation Therapy Oncology Group (RTOG) scale. Overall survival (OS) was defined as the time from admission to either death or time of analysis. Progression‐free survival (PFS) was calculated as the time from admission to either recurrence or death from any cause or time of analysis. Local‐regional failure‐free survival (LRFFS) was calculated as the time from admission to the time of local or regional failure. Distance metastasis free survival (DMFS) was calculated as the time from admission to the time of distant failure. Local failure included primary tumor and regional lymph node recurrence or progression. Distant failure included any site recurrence or progression beyond the primary tumor and regional lymph nodes. Overall response rate (ORR) was defined as the proportion of patients who achieved a complete or partial response in all evaluated patients. Disease control rate (DCR) was defined as the proportion of patients who achieved a complete or partial response or stable disease in all evaluated patients.

### 2.6 Statistical analysis

The data were analyzed using SPSS 13.0 software (International Business Machines Corporation (IBM), Armonk, New York, USA). The Kaplan‐Meier method was used for survival analysis. Log‐rank testing was performed to compare differences in outcome among the treatment groups. Fourteen predefined baseline variables were entered into the univariate analysis, including gender, KPS, BMI, smoking status, tumor length, tumor location, T stage, N stage, M stage, tumor TNM stage, radiotherapy techniques, radiation dose, concurrent chemotherapy, and tumor early response. Any variables reaching *P* = 0.10 were included in the multivariate analysis. Chi‐square test (*χ* ^2^ test) was used for comparison of nonparametric variables between groups. All the tests were two‐sided, and *P* < 0.05 was considered statistically significant.

## 3 RESULTS

In total, 271 patients were included in the present study. The last follow‐up was in January 2017. At the time of analysis, 88 patients remained alive.

### 3.1 Clinical Characteristics

The patient characteristics are shown in Table 1. Of this patient cohort, 43.5% (n = 118) underwent radiotherapy only (RT alone), 21.1% (n = 57) received CCRT‐1, and 35.4% (n = 96) received CCRT‐2. 3D‐CRT and IMRT were used in 38.7% and 32.5% of the patients, respectively. The middle thoracic esophagus was the most common tumor location (n = 132, 48.7%), followed by the cervical/upper thoracic esophagus (n = 112, 41.3%) and lower thoracic esophagus (n = 27, 10.0%). The tumor length ranged from 1 to 13.7 cm, with a mean length of 5.7 ± 0.1 cm. The majority of patients had stage III (37.3%) or IV (32.8%) tumors. There were 192 men and 79 women, and the mean age was 72.4 ± 5.4 years (range 65‐89 years). Upon admission, 216 patients had a BMI >18.5 kg/m^2^. A total of 146 of the 271 patients had a smoking history. 21.8% of the patients had a family history of cancer, and 39.1% had comorbidities, including hypertension (57), diabetes (20), coronary artery disease (eight), cerebrovascular disease (three), peptic ulcer disease (11), liver disease (six), chronic pulmonary disease (seven), and pulmonary tuberculosis (13). Baseline characteristics were well balanced among the groups in this study except for T stage and radiotherapy techniques.

**Table 1:** Clinical characteristics of elderly patients with esophageal cancer treated with RT alone or CCRT‐1, or CCRT‐2

Variable	Total (271)	RT alone (118)	CCRT‐1 (57)	CCRT‐2 (96)	*P* value
Age (y)					
Median (range)	72 (65‐89)	75 (65‐89)	73 (65‐86)	67 (65‐78)	
Gender (%)					
Male	192 (70.8)	82 (69.5)	40 (70.2)	70 (72.9)	0.807
Female	79 (29.2)	36 (30.5)	17 (29.8)	26 (27.1)	
Karnofsky performance status (%)					
<80	22 (8.1)	12 (10.2)	5 (8.8)	5 (5.2)	0.988
≥80	249 (91.9)	106 (89.8)	52 (91.2)	91 (94.8)	
BMI (kg/m^2^; %)					
≤18.5	51 (18.8)	27 (22.9)	10 (17.5)	14 (14.6)	0.642
>18.5	216 (79.7)	91 (77.1)	45 (78.9)	80 (83.3)	
Unknown	4 (1.5)	0 (0.0)	2 (3.6)	2 (2.1)	
Smoking status (%)					
No	125 (46.1)	59 (50.0)	29 (50.9)	37 (38.5)	0.202
Yes	146 (53.9)	59 (50.0)	28 (49.1)	59 (61.5)	
Tumor length (cm; %)					
≤5.7	126 (46.5)	49 (41.5)	32 (56.1)	45 (46.9)	0.339
>5.7	114 (42.1)	49 (41.5)	20 (35.1)	45 (46.9)	
Unknown	31 (11.4)	20 (17.0)	5 (8.8)	6 (6.2)	
Tumor location (%)					
Cervical	31 (11.4)	7 (5.9)	10 (17.5)	14 (14.6)	0.136
Thoracic	240 (88.6)	111 (94.1)	47 (82.5)	82 (85.4)	
T stage (%)					
T1‐2	44 (16.2)	13 (11.0)	19 (33.3)	12 (12.5)	**0.040**
T3‐4	189 (69.7)	75 (63.6)	35 (61.4)	79 (82.3)	
Unknown	38 (14.1)	30 (25.4)	3 (5.3)	5 (5.2)	
N stage (%)					
N0	55 (20.3)	25 (21.2)	14 (24.5)	16 (16.7)	0.239
N1	180 (66.4)	65 (55.1)	40 (70.2)	75 (78.1)	
Unknown	36 (13.3)	28 (23.7)	3 (5.3)	5 (5.2)	
M stage (%)					
M0	153 (56.5)	60 (50.8)	36 (63.2)	57 (59.4)	0.938
M1a	32 (11.8)	13 (11.1)	5 (8.8)	14 (14.6)	
M1b	57 (21.0)	20 (16.9)	15 (26.3)	22 (22.9)	
Unknown	29 (10.7)	25 (21.2)	1 (1.7)	3 (3.1)	
Tumor TNM stage (%)					
I + II	52 (19.2)	21 (17.8)	17 (29.8)	14 (14.6)	0.509
III	101 (37.3)	38 (32.2)	19 (33.3)	44 (45.8)	
IVa	32 (11.8)	13 (11.0)	5 (8.8)	14 (14.6)	
IVb	57 (21.0)	20 (16.9)	15 (26.3)	22 (22.9)	
Unknown	29 (10.7)	26 (22.0)	1 (1.8)	2 (2.1)	
Radiotherapy techniques (%)					
2D‐RT	78 (28.8)	41 (34.7)	6 (10.5)	31 (32.3)	**0.015**
3D‐CRT/IMRT	193 (71.2)	77 (65.3)	51 (89.5)	65 (67.6)	
Radiation dose (Gy; %)					
≤54	60 (22.1)	33 (28.0)	13 (22.8)	14 (14.6)	0.064
>54	211 (77.9)	85 (72.0)	44 (77.2)	82 (85.4)	
Tumour early response (%)					
CR/PR	176 (64.9)	61 (51.7)	37 (64.9)	78 (81.3)	**0.004**
SD/PD	75 (27.7)	40 (33.9)	18 (31.6)	17 (17.7)	
Unknown	20 (7.4)	17 (14.4)	2 (3.5)	1 (1.0)	
Comorbidities (%)					
No	165 (60.9)	71 (60.2)	33 (57.9)	61 (63.5)	0.770
Yes	106 (39.1)	47 (39.8)	24 (42.1)	35 (39.1)	
Family history of cancer (%)					
No	212 (78.2)	90 (76.3)	49 (86.0)	73 (76.0)	0.282
Yes	59 (21.8)	28 (23.7)	8 (14.0)	23 (24.0)	

The *P* value in bold indicated that the difference among RT alone, CCRT‐1 and CCRT‐2 were significant.

### 3.2 Overall survival

**Figure 1:**
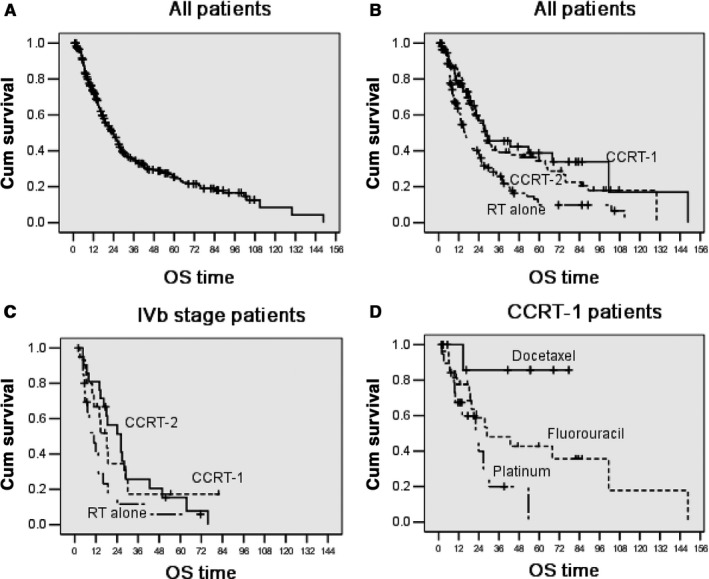
Overall survival of (A) all patients (n = 271), (B) patients treated with RT alone (n = 118), single‐agent CCRT (n = 56), and double‐agents CCRT (n = 97), (C) patients with stage IVb esophageal cancer (n = 57), (D) patients treated with docetaxel, fluorouracil, or platinum

The median survival time was 23.6 ± 2.3 months for all patients (Figure 1A). The 1‐, 2‐, and 5‐year survival rate was 73.0 ± 3.0%, 48.0 ± 3.0%, and 25.0 ± 3.0%, respectively (Table 2). Compared with the RT alone group, patients who underwent CCRT demonstrated improved survival benefits (*P* = 0.000) with a median survival time of 27.8 ± 2.4 and 15.6 ± 1.9 months, respectively. In the RT alone, CCRT‐1, and CCRT‐2 groups, the median OS was 15.6 ± 1.9 months, 28.8 ± 10.1 months, and 27.8 ± 2.5 months, respectively (Figure 1B). The 1‐year survival rate in the RT alone, CCRT‐1, and CCRT‐2 patients was 63.0 ± 5.0%, 78.0 ± 6.0%, and 82.0 ± 4.0%, respectively.

**Table 2:** Efficacy of treatments

Response	All (271)	RT alone (118)	CCRT‐1 (57)	CCRT‐2 (96)	*P* value
ORR (%)	70.1	60.3	67.2	82.1	0.004
DCR (%)	94.4	92.1	94.5	97.9	0.340
Median OS (mo)	23.6 ± 2.3	15.6 ± 1.9	28.8 ± 10.1	27.8 ± 2.5	0.000*
1‐y OS (%)	73.0 ± 3.0	63.0 ± 5.0	78.0 ± 6.0	82.0 ± 4.0	
2‐y OS (%)	48.0 ± 3.0	39.0 ± 5.0	59.0 ± 7.0	57.0 ± 5.0	
5‐y OS (%)	25.0 ± 3.0	11.0 ± 4.0	40.0 ± 8.0	34.0 ± 5.0	
Median PFS (mo)	13.6 ± 1.3	10.4 ± 0.9	16.5 ± 3.2	17.0 ± 2.0	0.020**
1‐y PFS (%)	54.0 ± 4.0	43.0 ± 4.0	56.0 ± 8.0	66.0 ± 10.0	
2‐y PFS (%)	33.0 ± 4.0	26.0 ± 5.0	39.0 ± 14.0	39.0 ± 9.0	

CCRT‐1, concurrent single‐agent‐based chemoradiotherapy; CCRT‐2, concurrent double‐agent‐based chemoradiotherapy; DCR, disease control rate; ORR, overall response rate; OS, overall survival; PFS, progression‐free survival; RT alone, radiotherapy alone.

* The *P* value between RT alone and CCRT‐1, CCRT‐2 was 0.001 and 0.000, respectively. The *P* value between CCRT‐1 and CCRT‐2 was 0.537.

** The *P* value between RT alone and CCRT‐1, CCRT‐2 was 0.290 and 0.005, respectively. The *P* value between CCRT‐1 and CCRT‐2 was 0.259.

Predictive factors of overall survival in the univariate analysis were gender, KPS, smoking status, tumor length, tumor location, T stage, M stage, tumor TNM stage, radiotherapy techniques, radiation dose, concurrent chemotherapy, and tumor early response (Table 3). In the multivariate analysis, T stage (*P* = 0.04), M stage (*P* = 0.00), radiotherapy techniques (*P* = 0.02), radiation dose (*P* = 0.00), concurrent chemotherapy (*P* = 0.04), and tumor early response (*P* = 0.01) were identified as independent prognostic factors of overall survival (Table 4).

**Table 3:** **U**nivariate analysis of prognostic factors on treatment results (n = 271)

Prognostic factors	OS		PFS		LRFFS		DMFS	
	*P*	HR (95% CI)	*P*	HR (95% CI)	*P*	HR (95% CI)	*P*	HR (95% CI)
Gender (male vs female )	**0.01**	1.56 (1.11‐2.20)	**0.07**	1.34 (0.98‐1.83)	**0.04**	1.54 (1.02‐2.33)	0.64	1.10 (0.72‐1.69)
KPS (<80 vs ≥80)	**0.09**	0.61 (0.35‐1.08)	0.13	0.66 (0.39‐1.12)	**0.07**	0.55 (0.29‐1.04)	0.98	0.98 (0.39‐2.43)
BMI (≤ 18.5 kg/m^2^ vs >18.5 kg/m^2^)	0.86	0.97 (0.66‐1.42)	0.46	1.15 (0.79‐1.67)	0.82	1.05 (0.66‐1.67)	0.49	1.21 (0.70‐2.07)
Smoking status (no vs yes)	**0.08**	1.31 (0.97‐1.76)	**0.03**	1.36 (1.03‐1.81)	**0.01**	1.58 (1.09‐2.28)	0.57	1.12 (0.75‐1.66)
Tumor length (≤5.7 cm vs >5.7 cm)	**0.02**	1.46 (1.08‐1.98)	0.12	1.26 (0.94‐1.68)	0.16	1.30 (0.90‐1.88)	0.14	1.35 (0.90‐2.02)
Tumor location (cervical vs thoracic)	**0.08**	1.51 (0.95‐2.41)	0.31	1.26 (0.81‐1.94)	0.55	1.18 (0.68‐2.02)	0.36	1.33 (0.71‐2.50)
T stage (T1‐2 vs T3‐4)	**0.02**	1.79 (1.12‐2.87)	**0.03**	1.58 (1.04‐2.40)	**0.02**	2.00 (1.12‐3.59)	0.28	1.35 (0.78‐2.35)
N stage (N0 vs N1)	0.65	1.09 (0.76‐1.56)	0.36	1.17 (0.83‐1.65)	0.72	1.08 (0.70‐1.66)	0.12	1.49 (0.90‐2.48)
M stage (M0 vs M1)	**0.00**	1.92 (1.39‐2.64)	**0.00**	1.78 (1.32‐2.41)	**0.00**	2.16 (1.47‐3.17)	**0.02**	1.67 (1.09‐2.56)
Tumor TNM stage (I + II vs III+IV)	**0.01**	1.71 (1.15‐2.56)	**0.01**	1.63 (1.13‐2.35)	**0.01**	2.07 (1.25‐3.44)	0.26	1.31 (0.81‐2.12)
Radiotherapy techniques (2D‐RT vs 3D‐CRT/IMRT)	**0.00**	0.60 (0.45‐0.81)	0.10	0.77 (0.57‐1.04)	0.38	0.85 (0.57‐1.23)	**0.05**	0.67 (0.44‐1.00)
Radiation dose (≤54 Gy vs >54 Gy)	**0.00**	0.47 (0.34‐0.66)	**0.00**	0.47 (0.34‐0.64)	**0.00**	0.43 (0.29‐0.63)	**0.02**	0.56 (0.35‐0.90)
Concurrent chemotherapy (none vs single agent vs double agents)	**0.01**	0.78 (0.65‐0.94)	**0.01**	0.79 (0.68‐0.93)	**0.03**	0.79 (0.65‐0.97)	**0.08**	0.81 (0.65‐1.02)
Tumor early response (CR/PR vs SD/PD)	**0.01**	1.50 (1.09‐2.07)	**0.02**	1.44 (1.06‐1.96)	**0.07**	1.42 (0.96‐2.11)	0.31	1.25 (0.81‐1.95)

DMFS, distance metastasis free survival; LRFFS, local‐regional failure‐free survival; OS, overall survival; PFS, progression‐free survival.

The *P* value in bold indicated that the prognostic factor was associated with OS, PFS, LRFFS or DMFS.

**Table 4:** Multivariate analysis of prognostic factors on treatment results (n = 271)

Endpoint	Prognostic factors	Multivariate analysis	
		*P*	HR (95% CI)
OS	T stage (T1‐2 vs T3‐4)	0.04	1.73 (1.03‐2.90)
	M stage (M0 vs M1)	0.00	2.08 (1.49‐2.93)
	Radiotherapy techniques (2D‐RT vs 3D‐CRT/IMRT)	0.02	0.67 (0.47‐0.94)
	Radiation dose (≤54 Gy vs >54 Gy)	0.00	0.53 (0.37‐0.77)
	Concurrent chemotherapy (none vs single agent vs double agents)	0.04	0.82 (0.68‐0.99)
	Tumor early response (CR/PR vs SD/PD)	0.01	1.60 (1.12‐2.30)
PFS	M stage (M0 vs M1)	0.00	1.76 (1.29‐2.41)
	Concurrent chemotherapy (none vs single agent vs double agents)	0.01	0.80 (0.67‐0.95)
	Radiation dose (≤54 Gy vs >54 Gy)	0.00	0.55 (0.39‐0.78)
LRFFS	M stage (M0 vs M1)	0.00	1.99 (1.34‐2.95)
	Radiation dose (≤54 Gy vs >54 Gy)	0.00	0.49 (0.31‐0.75)
DMFS	M stage (M0 vs M1)	0.02	1.70 (1.10‐2.62)
	Concurrent chemotherapy (none vs single agent vs double agents)	0.04	0.78 (0.62‐0.99)

DMFS, distance metastasis free survival; LRFFS, local‐regional failure‐free survival; OS, overall survival; PFS, progression‐free survival.

For patients with IVb stage disease, the median OS was slightly better in the CCRT‐2 group than in the CCRT‐1 group and RT alone group. The difference between the CCRT‐2 and RT alone group was significant (*P* = 0.027) but not the difference between the CCRT‐1 and RT alone group (*P* = 0.123; Figure 1C). Among patients who received CCRT‐1, we further evaluated the efficacy of different chemotherapy regimens. Patients in the CCRT‐1 group were divided into the following three groups: those who received docetaxel (n = 11), fluorouracil (n = 19), or platinum (n = 27). As shown in Figure 1D, the OS in the three groups was significantly different (*P* = 0.017).

### 3.3 Progression‐free survival

**Figure 2:**
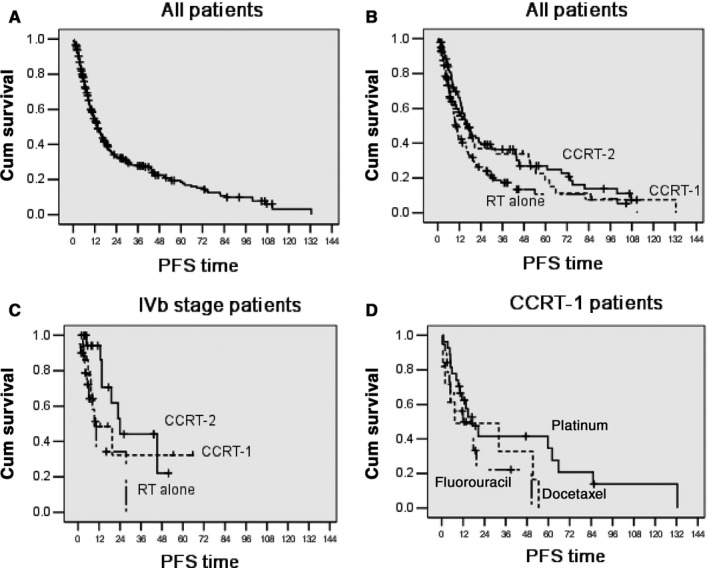
Progression‐free survival of (A) all patients (n = 271), (B) patients treated with RT alone (n = 118), single‐agent CCRT (n = 56), and double‐agents CCRT (n = 97), (C) patients with stage IVb esophageal cancer (n = 57), (D) patients treated with docetaxel, fluorouracil, or platinum

The 1‐, 2‐, and 5‐year PFS rate of the entire cohort was 54.0 ± 4.0%, 33.0 ± 4.0%, and 19.0 ± 12.0%, respectively, with a median PFS time of 13.6 ± 1.3 months (Figure 2A). The progression‐free survival time of patients who underwent CCRT and RT alone was 16.5 ± 1.9 months and 10.4 ± 0.9 months, respectively (*P* = 0.004). Among the 118 patients who received RT alone, the 1‐ and 5‐year PFS rate was 43.0 ± 4.0% and 12.0 ± 13.0%, respectively, with a median PFS time of 10.4 ± 0.9 months. The median PFS time was 16.5 ± 3.2 months in CCRT‐1 patients and 17.0 ± 2.0 months in CCRT‐2 patients (*P* = 0.321). The difference in PFS was significant between the CCRT‐2 and RT alone group, but not between the CCRT‐1 and RT alone group (Figure 2B). The 1‐year and 2‐year PFS rate in CCRT‐1 patients was 56.0 ± 8.0% and 39.0 ± 14.0%, respectively, and 66.0 ± 10.0% and 39.0 ± 9.0% in CCRT‐2 patients, respectively.

Predictive factors of PFS in the univariate analysis were gender, smoking status, T stage, M stage, tumor TNM stage, radiation dose, concurrent chemotherapy, and tumor early response (Table 3). In multivariate analysis, M stage (*P* = 0.00), concurrent chemotherapy (*P* = 0.01) and radiation dose (*P* = 0.00) were identified as independent prognostic factors of progression‐free survival (Table 4).

For patients with IVb stage, PFS was slightly better in the CCRT‐2 group than that in the CCRT‐1 group and RT alone group. The difference between the CCRT‐2 and RT alone group was significant (*P* = 0.002) but not that between the CCRT‐1 and RT alone group (*P* = 0.089; Figure 2C). As shown in Figure 2D, the difference in PFS in the patients treated with docetaxel, fluorouracil, or platinum‐based concurrent chemoradiotherapy was not statistically significant (*P* = 0.140).

### 3.4 Early side effects

The AEs were summarized in Table 5. Most treatment‐related toxicities were grade 1 to 2. Grade ≥3 AEs occurred in 25.5% (n = 69/271) of the patients, with 10 in the RT alone group, 15 in the CCRT‐1 group, and 44 in the CCRT‐2 group (*P* = 0.000). Grade 3 or worse acute hematological toxicities were observed in 22.1% of patients. CCRT‐2 was more toxic than CCRT‐1. As shown in Table 4, grade ≥3 hematological toxicity was observed more frequently in the patients treated with CCRT‐2 than in the patients who received CCRT‐1 (42.7% vs 22.8%, *P* = 0.013) or patients who received RT alone (42.7% vs 5.1%, *P* = 0.000). The differences in leukocytopenia and weight loss during treatment for patients treated with CCRT‐1 or CCRT‐2 were statistically significant (*P* = 0.016 and *P* = 0.011, respectively).

**Table 5:** Adverse events due to radiotherapy alone, CCRT‐1, and CCRT‐2 in elderly patients with esophageal cancer

Variable	Total (%)	RT alone (%)	CCRT‐1 (%)	CCRT‐2 (%)	*P* value
Total adverse events					
Grade 0‐2	202/271 (74.5)	108/118 (91.5)	42/57 (73.7)	52/96 (54.2)	**0.000**
Grade 3‐4	69/271 (25.5)	10/118 (8.5)	15/57 (26.3)	44/96 (45.8)	
Hematologic toxicities					
Grade 0‐2	211/271 (77.9)	112/118 (94.9)	44/57 (77.2)	55/96 (57.3)	**0.000**
Grade 3‐4	60/271 (22.1)	6/118 (5.1)	13/57 (22.8)	41/96 (42.7)	
Leukocytopenia					
Grade 0‐2	219/270 (81.1)	118/118 (100)	44/56 (78.6)	57/96 (59.4)	**0.000**
Grade 3‐4	51/270 (18.9)	0/118 (0)	12/56 (21.4)	39/96 (40.6)	
Thrombocytopenia					
Grade 0‐2	251/268 (93.7)	114/117 (97.4)	53/55 (96.4)	84/96 (87.5)	**0.008**
Grade 3‐4	17/268 (6.3)	3/117 (2.6)	2/55 (3.6)	12/96 (12.5)	
Anemia					
Grade 0‐2	260/268 (97)	113/116 (97.4)	55/56 (98.2)	92/96 (95.8)	0.670
Grade 3	8/268 (3)	3/116 (2.6)	1/56 (1.8)	4/96 (4.2)	
Hypoalbuminemia					
Grade 1‐2	263/263 (100)	114/114 (100)	56/56 (100)	93/93 (100)	1.000
Grade 3	0/263 (0)	0/114 (0)	0/56 (0)	0/93 (0)	
Weight loss during treatment					
Grade 0‐1	235/268 (87.7)	103/117 (88.0)	54/56 (96.4)	78/95 (82.1)	**0.035**
Grade 2‐3	33/268 (12.3)	14/117 (12.0)	2/56 (3.6)	17/95 (17.9)	
Gastrointestinal reaction					
Grade 0‐1	228/264 (86.4)	103/113 (91.2)	48/56 (85.7)	77/95 (81.1)	0.107
Grade 2‐4	36/264 (13.6)	10/113 (8.8)	8/56 (14.3)	18/95 (18.9)	
Esophagitis					
Grade 0‐1	183/265 (69.1)	90/113 (79.6)	38/56 (67.9)	55/96 (57.3)	**0.002**
Grade 2‐4	82/265 (30.9)	23/113 (20.4)	18/56 (32.1)	41/96 (42.7)	
Lung fibrosis					
Grade 0‐1	258/264 (97.7)	112/113 (99.1)	54/56 (96.4)	92/95 (96.8)	0.420
Grade 2‐4	6/264 (2.3)	1/113 (0.9)	2/56 (3.6)	3/95 (3.2)	

The *P* value in bold indicated that the incident rate of adverse event among RT alone, CCRT‐1 and CCRT‐2 was significant.

### 3.5 Early responses

All patients were evaluated for early response at the end of treatment. The CR, PR, SD, and PD rates in each group were listed in Table 1. The ORR in RT alone, CCRT‐1, and CCRT‐2 groups was 60.3%, 67.2%, and 82.1%, respectively (*P* = 0.004). The DCR in the RT alone, CCRT‐1, and CCRT‐2 groups was 92.1%, 94.5%, and 97.9%, respectively (*P* = 0.34; Table 2). The ORR and DCR in the CCRT group were 76.7% and 96.0%, respectively.

### 3.6 Treatment failure

At the time of analysis, 201 patients had suffered from disease progression based on the available information. In those patients, 101 had local failures, 76 had distant failures, and 21 had both local and distant failures. The predictive factors for local and regional failure‐free survival in the multivariate analysis were M stage (*P* = 0.00) and radiation dose (*P* = 0.00). The predictive factors for distant failure‐free survival in the multivariate analysis were M stage (*P* = 0.02) and concurrent chemotherapy (*P* = 0.04; Table 4). There were 166 deaths after recurrence. The most common sites of distant metastases were lung and bone.

## 4 DISCUSSION

The aging of the population combined with an increased life expectancy has led to more elderly patients with cancer being referred for treatment. Our retrospective data suggested that CCRT in elderly patients with OC resulted in better ORR and DCR, as well as improved survival compared with RT alone. Moreover, our data showed that compared with patients treated with CCRT‐2, elderly patients treated with CCRT‐1 experienced similar survival but less severe toxicities. Further analysis of patients who received CCRT‐1 indicated that patients treated with docetaxel displayed a better survival outcome in OS but not in PFS than patients treated with fluorouracil or platinum.

Chemoradiation is the standard nonsurgical treatment for patients with locally advanced OC.^11^, ^12^ The role of CCRT in elderly OC patients remains unclear because patients older than 70 years were always excluded from most randomized trials. A review of the American National Cancer Database revealed that definitive chemoradiotherapy was a treatment option for inoperable patients older than 70 years and led to a median survival of 15.3 months.^13^ The results of a phase II, single‐arm study evaluating platinum‐based chemotherapy combined with radiotherapy in patients older than 75 years with esophageal carcinoma suggested that chemotherapy is feasible and tolerable in elderly patients.^14^ Meanwhile, there have been several retrospective studies^14^, ^15^, ^16^, ^17^, ^18^, ^19^, ^20^, ^21^, ^22^, ^23^, ^24^ in elderly patients with OC had indicated that concurrent chemoradiation might be an optimal strategy for elderly patients with OC. Studies with more than 20 patients were listed in Table 6. In those studies, survival showed great variation, with the median survival time ranging from 9 to 35 months, the 1‐year OS ranging from 39% to 98%, and the 2‐year OS ranging from 27% to 78%. Several factors might account for this discrepancy. First, most of the studies were small‐sample‐sized retrospective studies, and thus, selection bias could exist. Second, the radiotherapy techniques were different, some studies used 2D conventional radiotherapy, while others used 3D‐CRT. Third, the age of patients differed. Finally, the chemotherapy regiments were diverse. All of the abovementioned factors substantially affected the clinical outcome. In our study, a total of 271 OC patients older than 65 years from two centers were enrolled. Compared with patients treated with RT alone, patients who received CCRT had better survival outcomes (OS, 27.8 ± 2.4 vs 15.6 ± 1.9; PFS, 16.5 ± 1.9 vs 10.4 ± 0.9 months). Our results in elderly patients were consistent with those of previous studies,^15^, ^17^, ^18^, ^24^ but our study is the first to compare the efficacy of CCRT‐1 and CCRT‐2 in patients. However, the data failed to identify a statistically significant difference in median OS or median PFS. In analysis of patients with IVb stage cancer, the OS and PFS were significantly different between patients who received CCRT‐2 and RT alone but not between patients treated with CCRT‐1 and RT alone. The data showed that CCRT‐2 might be a better choice for patients with stage IVb OC. Furthermore, the ORR and DCR in the CCRT group were 76.7% and 96.0%, respectively, which were higher than those in the RT alone group (*P* = 0.006 and *P* = 0.184, respectively). These results suggested that CCRT is valuable for elderly OC patients. The results could be explained by radio‐sensitization. Chemotherapy may enhance the local effects of radiation and thus decrease the probability of spread from the primary tumor and reduce micrometastases.

**Table 6:** Previous studies of radiotherapy with or without chemotherapy for elderly patients with esophageal squamous cell carcinoma

Study	N	Age	Stage	Treatment (n)	Median OS (mo)	1y‐OS **(%)**	2y‐OS **(%)**	≥3 grade AEs **(%)**	≥3 grade hematologic toxicity **(%)**
Chen (2017)^15^	90	≥65	II‐III	CCRT with XP (49)	30.6	98^a^	78^a^	26.5	
				RT alone (41)	18.7	96^a^	20^a^	20.6	
Qu (2015)^16^	74	≥70		Curative treatment (46) b	18.6	70	37		
				Not‐curative treatment (26)	8.8	37	10	12	
Zhang (2014)^17^	128	≥65	I‐IV	CCRT (73)	22	91^a^	57^a^		36.9
				RT alone (55)	13	62^a^	42^a^		14.5
Song (2015)^18^	82	≥70	I‐IV	CCRT with TP	26.9				30.4
Li (2015)^24^	116	≥70	I‐IV	CCRT (32)	22.3	70^a^	50^a^	25	
				sCRT (24)	18.0	68^a^	38^a^	16.7	
				RT alone (60)	12.4	52^a^	30^a^	13.3	
Tougeron (2008)^19^	109	≥70	I‐IV	CCRT	15.2	58^a^	35.5	23.8	
Servagi‐Vernat (2015)^14^	30	≥75	II‐III	CCRT with cisplatin or oxaliplatin	14.5	55	27		
Mak (2010)^20^	34	≥75	I‐IV	CCRT	12.4		29.7	73.5	35.2
Anderson SE (2007)^21^	25	≥65	II‐III	CCRT with 5‐FU and mitomycin‐C	35	80	64		36
Uno (2004)^22^	22	≥75	I‐IV	CCRT with PF	9	39	18^a^		9
Nallapareddy (2005)^23^	30	≥70	I‐IV	CCRT	10	42^a^	29^a^		16.7
Current study	271	≥65	I‐IV	RT alone	15.6	63	39	8.5	5.1
				CCRT‐1	28.8	78	59	26.3	22.8
				CCRT‐2	27.8	82	57	45.8	42.7

CCRT, concurrent chemoradiotherapy; CCRT‐1, single‐agent‐based concurrent chemoradiotherapy; CCRT‐2, double‐agent‐based concurrent chemoradiotherapy; PF, cisplatin/carboplatin plus 5‐fluorouracil; RT alone, radiotherapy alone; sCRT, sequential chemoradiotherapy; TP, paclitaxel plus cisplatin; XP, capecitabine plus cisplatin.

a Estimating from the survival curve.

b Including 40 patients treated with CCRT.

The optimal dose for esophageal squamous cell carcinoma was still controversial. Minsky et al^25^ studied 218 esophageal carcinoma patients receiving high‐dose (64.8 Gy) or standard‐dose (50.4 Gy) radiotherapy, and the results showed that no significance difference was seen for median survival and local/regional control between the two groups (RTOG 94‐05). However, Xu et al^26^ study indicated that patients received high‐dose (>50 Gy) led to a improved survival compared with patients received low‐dose (≤50 Gy). Our results found the Radiation dose >54 Gy was associated with better OS, PFS, and LRFFS (Table 4). The authors postulated several possible reasons for the difference, including: (a) The enrolled patients in RTOG 94‐05 had esophageal adenocarcinoma while most of the Asian OC patients had squamous cell carcinoma; (b) the radiotherapy techniques used in RTOG 94‐05 was 2D‐RT, and in our study, more than 70% patients irradiated with 3D‐RT/IMRT; (c) 60% patients also treated with concurrent chemotherapy in patients irradiated with a dose of >54 Gy, compared 45% in patients irradiated with a dose of ≤54 Gy. However, Well‐designed, larger multicenter prospective trials may be need to confirm the relationship between radiation dose and survival for Asian esophageal squamous carcinoma.

Although promising results were found for CCRT in elderly patients, older patients are at an increased risk for chemotherapy and radiotherapy toxicity.^27^ As shown in Table 6, the incidence of severe hematological toxicity in patients treated with concurrent chemoradiotherapy ranged from 9% to 36.5%. In our study, the rate of grade 3 or 4 hematological toxicity in the CCRT group was significantly higher than that in the RT alone group (35.3% vs 5.1%). Thus, it is important to reduce the toxicities for patients who received CCRT. We compared the treatment toxicities of CCRT‐1 and CCRT‐2 in elderly patients with OC. As expected, compared with CCRT‐2 patients, CCRT‐1 patients experienced a reduced rate of grade 3 or 4 hematological toxicities (42.7% vs 22.8%, respectively). The results might be due to the number of chemotherapy drugs associated with an increased risk of toxicity in the elderly population with cancer,^28^ and aging was associated with decreased bone marrow reserve and an increased risk of myelosuppressive‐associated complications from chemotherapy.^29^, ^30^ Receiving poly‐chemotherapy may further enhance the myelosuppressive effect.

Fluorouracil, platinum and docetaxel are agents commonly used to treat OC. In our study, we compared the survival benefit and toxicities among single agents, including fluorouracil, platinum or docetaxel plus radiotherapy. A better OS but not PFS was observed in the patients treated with docetaxel compared to that in the patients who received fluorouracil or platinum. The median OS of patients treated with docetaxel or fluorouracil was higher than that reported in Zhang's study^17^ (docetaxel, did not reach the median OS vs 21 months; fluorouracil, 22 vs 17 months). Zhu et al^31^ did a randomized phase 2 trial comparing definitive concurrent chemoradiotherapy with docetaxel plus cisplatin vs 5‐Fluorouracil plus cisplatin in patients with esophageal squamous cell carcinoma and reported no significant differences in overall survival and progression‐free survival between the two groups, but the docetaxel plus cisplatin regimen was associated with more severe hematological toxicities. Several factors may account for this discrepancy. First, the study cohort in our study was elder patients (≥75 years), while only 40% older than 60 years in Zhu's study. Second, we compared the treatment effects among single agent (fluorouracil, platinum, or docetaxel) plus radiotherapy, but zhu compared that between double agents plus radiotherapy, which might lead to the difference. Third, only 57 patients received CCRT‐1 in our study, including 19 treated with fluorouracil, 27 treated with platinum, and 11 treated with docetaxel, the sample size was small, and the selection bias was exist. Fourth, 90.9% patients who received docetaxel had no comorbidities, compared with 57.9% in patients received fluorouracil and 44.4% in patients received platinum, which was known to be prognostic impact. Fifth, more patients received better radiotherapy techniques applied (IMRT) in docetaxel cohort (72.7%) than that in fluorouracil (63.2%) or platinum (37.0%) cohort. Our data suggested that patients who tolerated docetaxel could achieve better survival results.

Our results from this trial supported the results of previous retrospective studies and demonstrated that CCRT was effective and tolerable in elderly patients with OC. Furthermore, we found that compared with patients treated with CCRT‐2, patients who received CCRT‐1 exhibited a reduced rate of treatment toxicities, but not at the expense of the survival benefit. Although the results are promising, but we must note that this was a retrospective study and the chemotherapy agents, doses and cycles were heterogeneous. Therefore, prospective clinical trials for elderly patients with OC are necessary.

## ETHICAL APPROVAL

All procedures performed in studies involving human participants conformed to the standards set by the Declaration of Helsinki and approved by the Human Ethics Committee of Sun Yat‐sen University Cancer Center. All the patients consent to participate in the study and signed the informed consent. All of the patient data were confidential.
